# The next frontier for genomic prediction is computational

**DOI:** 10.1093/g3journal/jkaf290

**Published:** 2026-02-04

**Authors:** Lauren M McIntyre

**Affiliations:** Department of Molecular Genetics and Microbiology, University of Florida Genetics Institute, University of Florida, PO Box 100266, Gainesville, FL 32610, United States

## Abstract

From the seminal paper by Meeuwissen *et al*. 2001 to the GSA Journals Series launch in 2015, the field of Genomic Prediction continues to gain momentum. The field is increasingly dynamic, with new technology increasing the scale and scope of the data available. Significant challenges exist in building computational models. Questions of how to appropriately account for different types of data, and which data improve predictions are interwoven. What is the best path forward? What methods will improve predictions? Authors can submit their rejoinder or start a new discussion on one of the many important topics in the field by submitting a Dialogue and Debate article for peer review at G3.

From the seminal paper by Meuwissen et al. (2001) to the GSA Journals Series launch in 2015, the field of Genomic Prediction continues to gain momentum. The field is increasingly dynamic, with new technology increasing the scale and scope of the data available. Significant challenges exist in building computational models. Questions of how to appropriately account for different types of data and which data improve predictions are interwoven. Recent papers at the GSA journals have endeavored to address relatedness and population structure (Pocrnic et al. 2024), historical data (Costa-Neto et al. 2023; Crossa et al. 2025; Vitale et al. 2025), phenotypic data from satellite images (Morales et al. 2024 ), hyperspectral imaging (Concepcion et al. 2025), physiological models, environmental interactions and variation in management practice, genome interactions (Yang et al. 2023 ), genome and environment interactions (Xavier et al. 2024), missing data, copy number/ploidy (Osorio-Guarin et al. 2024; Wilson et al. 2024; Endelman 2025; Tessele et al. 2025), low-input breeding potential (Olsson et al. 2025 ), and the genetic architecture of the trait of interest (Gibbs et al. 2025) into increasingly complex models.

What is the best path forward? What methods will improve predictions? Deep learning (Montesinos-López et al. 2023, 2024a, 2024b; Kihlman et al. 2024)? Regularization (Montesinos-López et al. 2024a, 2024b)? Ensemble models (Tomura et al. 2025)? Or something simpler (Ahlinder et al. 2024)? Fundamentally, we are all engaged with trying to understand how we can best predict beyond the environmental data at hand (Hu et al. 2025) and how important it is to have similar environments in the training data (Rogers and Holland 2022).

Howard and Lipka (2025) argue that we should consider simpler models in “Genomic selection and reproducibility: are complex models distracting us from true scientific validity in the presence of genotype-by-environment interaction,” the latest Dialogue and Debate published in G3. Do you share this perspective? You can submit your rejoinder or start a new discussion on one of the many important topics in the field by submitting your Dialogue and Debate article for peer review at G3.
